# Supplementary material for: Robust small area estimation for unit level model with density power divergence
Source: PLoS One. 2023 Nov 16;18(11):e0288639. doi: 10.1371/journal.pone.0288639 (PMC10653428; doi:10.1371/journal.pone.0288639)
Supplement: S1 File — (PDF) [file pone.0288639.s001.pdf]

Robust Small Area Estimation for Unit Level Model with Density Power Divergence

Xijuan Niu[1,2,*], Zhiqiang Pang[1], Zhaoxu Wang[1,2]

1 Department of Statistics, Lanzhou University of Finance and Economics, Lanzhou, Gansu, China
2 Department of Statistics Mathematics and Statistics, Qinghai Normal University, Xining, Qinghai, China

* correspondingauthor: 2014107@qhnu.edu.cn

Supporting information

S1 Fig. Plot of the MSE of robust estimated parameters versus the contaminated proportion of e_{ij}. Left-hand panel, β_0, right-hand panel, β_1. RML, the robust estimation method presented in [24]; ML, maximum likelihood estimation; Mdpd1, Mdpd2 and Mdpd3 represent the minimum density power devergence method with tuning parameter $\gamma = 0.1, 0.2, 0.3$ respectively.

S2 Fig. The MSE of robust estimated parameters versus the contaminated proportion of e_{ij}. Left-hand panel, σ_e^2, right-hand panel, σ_v^2.

S3 Fig. The MSE of robust estimated parameters versus the contaminated proportion of v_i. Left-hand panel, β_0, right-hand panel, β_1.

S4 Fig. Plot of the MSE of robust estimated parameters versus the contaminated proportion of v_i. Left-hand panel, σ_e^2, right-hand panel, σ_v^2.

S5 Fig. The MSE of robust estimated parameters versus the contaminated proportion of (v_i, e_{ij}). Left-hand panel, β_0, right-hand panel, β_1.

S6 Fig. The MSE of robust estimated parameters versus the contaminated proportion of (v_i, e_{ij}). Left-hand panel, σ_e^2, right-hand panel, σ_v^2.

S7 Fig. The MSE of robust estimated parameters versus the contamination variance of e_{ij}. Left-hand panel, β_0, right-hand panel, β_1.

S8 Fig. Plot of the MSE of robust estimated parameters versus the contamination variance of e_{ij}. Left-hand panel, σ_e^2, right-hand panel, σ_v^2.

S9 Fig. The MSE of robust estimated parameters versus the contamination variance of v_i. Left-hand panel, β_0, right-hand panel, β_1.

S10 Fig. The MSE of robust estimated parameters versus the contamination variance of v_i. Left-hand panel, σ_e^2, right-hand panel, σ_v^2.

S11 Fig. The MSE of robust estimated parameters versus the contamination variance of (v_i, e_{ij}). Left-hand panel, β_0, right-hand panel, β_1.

S12 Fig. The MSE of robust estimated parameters versus the contamination variance of (v_i, e_{ij}). Left-hand panel, σ_e^2, right-hand panel, σ_v^2.

A Proof of Theorem 1

The following partial derivatives are used to solve the MDPDE estimation equation,

$$\frac{\partial|\mathbf{V}_i|}{\partial\boldsymbol{\beta}} = 0, \qquad \frac{\partial|\mathbf{V}_i|}{\partial\sigma_v^2} = a_{i\cdot}\sigma_e^{2(n_i-1)}\prod_{j=1}^{n_i} k_{ij}^2,$$

$$\frac{\partial|\mathbf{V}_i|}{\partial\sigma_e^2} = \left(n_i\sigma_e^{2(n_i-1)} + (n_i-1)a_{i\cdot}\sigma_v^2\sigma_e^{2(n_i-2)}\right)\prod_{j=1}^{n_i} k_{ij}^2,$$

$$\frac{\partial B_i}{\partial\boldsymbol{\beta}} = -\frac{2}{\sigma_e^2}\sum_{j=1}^{n_i} a_{ij}(y_{ij}-\mathbf{x}_{ij}\boldsymbol{\beta})\mathbf{x}_{ij}^T + \frac{2\sigma_v^2}{\sigma_e^2(\sigma_v^2+\frac{\sigma_e^2}{a_{i\cdot}})}\bar{\boldsymbol{x}}_i^T\left(\sum_{j=1}^{n_i} a_{ij}(y_{ij}-\mathbf{x}_{ij}\boldsymbol{\beta})\right),$$

$$\frac{\partial B_i}{\partial\sigma_v^2} = -\frac{1}{a_{i\cdot}^2(\sigma_v^2+\frac{\sigma_e^2}{a_{i\cdot}})^2}\left(\sum_{j=1}^{n_i} a_{ij}(y_{ij}-\mathbf{x}_{ij}\boldsymbol{\beta})\right)^2,$$

$$\frac{\partial B_i}{\partial\sigma_e^2} = -\frac{1}{\sigma_e^4}\sum_{j=1}^{n_i} a_{ij}(y_{ij}-\mathbf{x}_{ij}\boldsymbol{\beta})^2 + \frac{2\sigma_e^2\sigma_v^2 + a_{i\cdot}\sigma_v^4}{(a_{i\cdot}\sigma_v^2+\sigma_e^2)^2\sigma_e^4}\left(\sum_{j=1}^{n_i} a_{ij}(y_{ij}-\mathbf{x}_{ij}\boldsymbol{\beta})\right)^2,$$

Proof. The proof of the first part closely follows the consistency of the maximum likelihood estimator with the line of modifications as given in Theorem 3.1 of [28]. For brevity, we only present the detailed proof of the second part. Let $\widehat{\boldsymbol{\theta}}$ be the MDPDE of $\boldsymbol{\theta}$. Then

$$\frac{\partial}{\partial\boldsymbol{\theta}}\widehat{d}_\gamma\left(f_{\boldsymbol{\theta}}, g\right) = \frac{\partial}{\partial\boldsymbol{\theta}}\left[\frac{1}{m}\sum_{i=1}^m\int_y f_{\boldsymbol{\theta}}^{1+\gamma}\left(y\mid\mathbf{x}_i\right)dy - \frac{1+\gamma}{m\gamma}\sum_{i=1}^m f_{\boldsymbol{\theta}}^{\gamma}\left(y_i\mid\mathbf{x}_i\right)\right] = 0$$

Thus, it can be written as the estimating equation of an M-estimator as follows

$$\sum_{i=1}^m \Psi_{\widehat{\boldsymbol{\theta}}}\left(y_i\mid\mathbf{x}_i\right) = 0. \tag{1}$$

where

$$\Psi_{\boldsymbol{\theta}}\left(y_i\mid\mathbf{x}_i\right) = u_{\boldsymbol{\theta}}\left(y_i\mid\mathbf{x}_i\right)f_{\boldsymbol{\theta}}^{\gamma}\left(y_i\mid\mathbf{x}_i\right) - \int_y u_{\boldsymbol{\theta}}\left(y\mid\mathbf{x}_i\right)f_{\boldsymbol{\theta}}^{1+\gamma}\left(y_i\mid\mathbf{x}_i\right)dy$$

Let $\boldsymbol{\theta}_g$ be the true value of $\boldsymbol{\theta}$, then $\mathrm{E}\left(\sum_{i=1}^m \Psi_{\widehat{\boldsymbol{\theta}}}\left(y_i\mid\mathbf{x}_i\right)\right) = 0$ gives

$$\sum_{i=1}^m\left[\int_y u_{\boldsymbol{\theta}_g}\left(y\mid\mathbf{x}_i\right)f_{\boldsymbol{\theta}_g}^{\gamma}\left(y\mid\mathbf{x}_i\right)g\left(y\mid\mathbf{x}_i\right)dy - \int_y u_{\boldsymbol{\theta}_g}\left(y\mid\mathbf{x}_i\right)f_{\boldsymbol{\theta}_g}^{1+\gamma}\left(y_i\mid\mathbf{x}_i\right)dy\right] = 0. \tag{2}$$

Taking a Taylor series expansion of Equation (1), we get

$$\frac{1}{m}\sum_{i=1}^{m}\Psi_{\boldsymbol{\theta}_g}\left(y_i \mid \mathbf{x}_i\right)+\frac{1}{m}\sum_{i=1}^{m}\frac{\partial}{\partial\boldsymbol{\theta}}\Psi_{\boldsymbol{\theta}}\left(y_i \mid \mathbf{x}_i\right)\bigg|_{\boldsymbol{\theta}=\boldsymbol{\theta}_g}\left(\widehat{\boldsymbol{\theta}}-\boldsymbol{\theta}_g\right)+R_m=0$$

$$\textbf{or } \sqrt{m}\left(\widehat{\boldsymbol{\theta}}-\boldsymbol{\theta}_g\right)=-\left[\frac{1}{m}\sum_{i=1}^{m}\frac{\partial}{\partial\boldsymbol{\theta}}\Psi_{\boldsymbol{\theta}}\left(y_i \mid \mathbf{x}_i\right)\bigg|_{\boldsymbol{\theta}=\boldsymbol{\theta}_g}\right]^{-1}\left[\frac{1}{\sqrt{m}}\sum_{i=1}^{m}\Psi_{\boldsymbol{\theta}_g}\left(y_i \mid \mathbf{x}_i\right)+\sqrt{m}R_m\right]. \tag{3}$$

where R_N is the remainder term. Using the weak law of large numbers (WLLN), we have

$$\begin{aligned}
&\frac{1}{m}\sum_{i=1}^{m}\frac{\partial}{\partial\boldsymbol{\theta}}\Psi_{\boldsymbol{\theta}}\left(y_i \mid \mathbf{x}_i\right)\\
&\xrightarrow{p}\lim_{m\to\infty}\mathrm{E}\left[\frac{1}{m}\sum_{i=1}^{m}\frac{\partial}{\partial\boldsymbol{\theta}}\Psi_{\boldsymbol{\theta}}\left(y_i \mid \mathbf{x}_i\right)\right]\\
&\xrightarrow{p}\lim_{m\to\infty}\frac{1}{m}\sum_{i=1}^{m}\mathrm{E}\left[\frac{\partial}{\partial\boldsymbol{\theta}}\left(u_{\boldsymbol{\theta}}f_{\boldsymbol{\theta}}^{\gamma}-\int u_{\boldsymbol{\theta}}f_{\boldsymbol{\theta}}^{1+\gamma}\right)\right]\\
&\xrightarrow{p}\lim_{m\to\infty}\frac{1}{m}\sum_{i=1}^{m}\mathrm{E}\left[-I_{\boldsymbol{\theta}}f_{\boldsymbol{\theta}}^{\gamma}+\gamma u_{\boldsymbol{\theta}}u_{\boldsymbol{\theta}}^{T}f_{\boldsymbol{\theta}}^{\gamma}-\int\left\{-I_{\boldsymbol{\theta}}f_{\boldsymbol{\theta}}^{1+\gamma}+(1+\gamma)u_{\boldsymbol{\theta}}u_{\boldsymbol{\theta}}^{T}f_{\boldsymbol{\theta}}^{1+\gamma}\right\}\right]\\
&\xrightarrow{p}\lim_{m\to\infty}\frac{1}{m}\sum_{i=1}^{m}\left[-\int I_{\boldsymbol{\theta}}f_{\boldsymbol{\theta}}^{\gamma}g+\gamma\int u_{\boldsymbol{\theta}}u_{\boldsymbol{\theta}}^{T}f_{\boldsymbol{\theta}}^{\gamma}g+\int I_{\boldsymbol{\theta}}f_{\boldsymbol{\theta}}^{1+\gamma}-(1+\gamma)\int u_{\boldsymbol{\theta}}u_{\boldsymbol{\theta}}^{T}f_{\boldsymbol{\theta}}^{1+\gamma}\right]\\
&\xrightarrow{p}-\lim_{m\to\infty}\frac{1}{m}\sum_{i=1}^{m}\left[\int u_{\boldsymbol{\theta}}u_{\boldsymbol{\theta}}^{T}f_{\boldsymbol{\theta}}^{1+\gamma}+\int\left(I_{\boldsymbol{\theta}}-\gamma u_{\boldsymbol{\theta}}u_{\boldsymbol{\theta}}^{T}\right)\left(g-f_{\boldsymbol{\theta}}\right)f_{\boldsymbol{\theta}}^{\gamma}\right]
\end{aligned}$$

So

$$\frac{1}{m}\sum_{i=1}^{m}\frac{\partial}{\partial\boldsymbol{\theta}}\Psi_{\boldsymbol{\theta}}\left(y_i \mid \mathbf{x}_i\right)\bigg|_{\boldsymbol{\theta}=\boldsymbol{\theta}_g}\xrightarrow{p}-\lim_{m\to\infty}\frac{1}{m}\sum_{i=1}^{m}J^{(i)}=-J. \tag{4}$$

From Equation (2), we get

$$\begin{aligned}
&\mathrm{E}\left[\frac{1}{\sqrt{m}}\sum_{i=1}^{m}\Psi_{\boldsymbol{\theta}_g}\left(y_i \mid \mathbf{x}_i\right)\right]\\
&=\frac{1}{\sqrt{m}}\sum_{i=1}^{m}\left[\int_y u_{\boldsymbol{\theta}_g}\left(y \mid \mathbf{x}_i\right)f_{\boldsymbol{\theta}_g}^{\gamma}\left(y \mid \mathbf{x}_i\right)g\left(y \mid \mathbf{x}_i\right)dy-\int_y u_{\boldsymbol{\theta}_g}\left(y \mid \mathbf{x}_i\right)f_{\boldsymbol{\theta}_g}^{1+\gamma}\left(y_i \mid \mathbf{x}_i\right)dy\right]\\
&=0.
\end{aligned} \tag{5}$$

Then

$$\begin{aligned}
&V\left[\frac{1}{\sqrt{m}}\sum_{i=1}^{m}\Psi_{\boldsymbol{\theta}_g}\left(y_i \mid \mathbf{x}_i\right)\right]\\
&=\frac{1}{m}\sum_{i=1}^{m}V\left[\Psi_{\boldsymbol{\theta}_g}\left(y_i \mid \mathbf{x}_i\right)\right]\\
&=\frac{1}{m}\sum_{i=1}^{m}\left[\int_y u_{\boldsymbol{\theta}_g}\left(y \mid \mathbf{x}_i\right)u_{\boldsymbol{\theta}_g}^{T}\left(y \mid \mathbf{x}_i\right)f_{\boldsymbol{\theta}_g}^{2\gamma}\left(y \mid \mathbf{x}_i\right)g\left(y \mid \mathbf{x}_i\right)dy-\xi^{(i)}\xi^{(i)T}\right]
\end{aligned}$$

$$= \frac{1}{m} \sum_{i=1}^{m} K^{(i)}. \tag{6}$$

Following [37] and using Equations (5) and (6), the central limit theorem (CLT) for the independent but not identical random variables gives

$$\frac{1}{\sqrt{m}} \sum_{i=1}^{m} \Psi_{\boldsymbol{\theta}_g}\left(y_i \mid \mathbf{x}_i\right) \stackrel{a}{\sim} N(0, K). \tag{7}$$

Under regularity condition (3), it can be easily shown that the reminder term $\sqrt{m} R_m = o_p(1)$. Therefore, combining Equations (3) and (7), we get from Equation (3)

$$\sqrt{N}\left(\widehat{\boldsymbol{\theta}} - \boldsymbol{\theta}_g\right) \stackrel{a}{\sim} N\left(0, J^{-1} K J^{-1}\right)$$

This completes the proof.

B vector $\boldsymbol{\xi}^{(i)}$ at model

According to the definition of $\boldsymbol{\xi}^{(i)}, \mathbf{u}_{\boldsymbol{\theta}}$:

$$\boldsymbol{\xi}^{(i)} = \int_{\mathbf{y}} \mathbf{u}_{\boldsymbol{\theta}}\left(\mathbf{y} \mid \mathbf{x}_i\right) f_{\boldsymbol{\theta}}^{\gamma+1}\left(\mathbf{y} \mid \mathbf{x}_i\right) d\mathbf{y}$$

we get

$$\begin{aligned}
\boldsymbol{\xi}_{\boldsymbol{\beta}}^{(i)} &= \int_{\mathbf{y}_i} \mathbf{u}_{\boldsymbol{\beta}}\left(\mathbf{y}_i \mid \mathbf{x}_i\right) f_{\boldsymbol{\theta}}^{1+\gamma}\left(\mathbf{y}_i \mid \mathbf{x}_i\right) d\mathbf{y}_i \\
&= \int_{\mathbf{y}_i}\left[\frac{1}{\sigma_e^2} \sum_{j=1}^{n_i} a_{ij} x_{ij}\left(y_{ij} - x_{ij}\boldsymbol{\beta}\right) - \frac{a_{i\cdot}\sigma_v^2 \bar{\mathbf{x}}_i}{\sigma_e^2\left(\sigma_e^2 + a_{i\cdot}\sigma_v^2\right)} \sum_{j=1}^{n_i} a_{ij}\left(y_{ij} - x_{ij}\boldsymbol{\beta}\right)\right] f_{\boldsymbol{\theta}}^{1+\gamma}\left(\mathbf{y}_i \mid \mathbf{x}_i\right) d\mathbf{y}_i \\
&= 0, \text{ from (12).}
\end{aligned}$$

From Equations (??) and (??), we get

$$\begin{aligned}
\boldsymbol{\xi}_{\sigma_v^2}^{(i)} &= \int_{\mathbf{y}_i} \mathbf{u}_{\sigma_v^2}\left(\mathbf{y}_i \mid \mathbf{x}_i\right) f_{\boldsymbol{\theta}}^{1+\gamma}\left(\mathbf{y}_i \mid \mathbf{x}_i\right) d\mathbf{y}_i \\
&= \int_{\mathbf{y}_i}\left[-\frac{a_{i\cdot}}{2\left(\sigma_e^2 + a_{i\cdot}\sigma_v^2\right)} + \frac{1}{2\left(\sigma_e^2 + a_{i\cdot}\sigma_v^2\right)^2}\left\{\sum_{j=1}^{n_i} a_{ij}\left(y_{ij} - x_{ij}\boldsymbol{\beta}\right)\right\}^2\right] f_{\boldsymbol{\theta}}^{1+\gamma}\left(y_i \mid \mathbf{x}_i\right) d\mathbf{y}_i \\
&= -\frac{a_{i\cdot}}{2\left(\sigma_e^2 + a_{i\cdot}\sigma_v^2\right)} \times M(1+\gamma), \text{ using } (13) \\
&\quad + \frac{1}{2\left(\sigma_e^2 + a_{i\cdot}\sigma_v^2\right)^2} \times M\left(a_{i\cdot}\sigma_e^2 + a_{i\cdot}^2\sigma_v^2\right), \text{ using (14)} \\
&= -\frac{M a_{i\cdot}\gamma}{2\left(\sigma_e^2 + a_{i\cdot}\sigma_v^2\right)}
\end{aligned}$$

$$
\begin{aligned}
\boldsymbol{\xi}_{\sigma_e^2}^{(i)} &= \int_{\mathbf{y}_i} \mathbf{u}_{\sigma_e^2}\left(\mathbf{y}_i \mid \mathbf{x}_i\right) f_{\boldsymbol{\theta}}^{1+\gamma}\left(\mathbf{y}_i \mid \mathbf{x}_i\right) d\mathbf{y}_i \\
&= \int_{\mathbf{y}_i} \left[-\frac{n_i\sigma_e^2 + (n_i-1)a_{i\cdot}\sigma_v^2}{2\sigma_e^2\left(\sigma_e^2 + a_{i\cdot}\sigma_v^2\right)} + \frac{1}{2\sigma_e^4}\sum_{j=1}^{n_i} a_{ij}\left(y_{ij} - x_{ij}\beta\right)^2 \right. \\
&\qquad \left. -\frac{\sigma_v^2\left(2\sigma_e^2 + a_{i\cdot}\sigma_v^2\right)}{2\sigma_e^4\left(\sigma_e^2 + a_{i\cdot}\sigma_v^2\right)^2} \left\{\sum_{j=1}^{n_i} a_{ij}\left(y_{it} - x_{it}\boldsymbol{\beta}\right)\right\}^2 \right] f_{\boldsymbol{\theta}}^{1+\gamma}\left(\mathbf{y}_i \mid \mathbf{x}_i\right) d\mathbf{y}_i \\
&= -\frac{n_i\sigma_e^2 + (n_i-1)a_{i\cdot}\sigma_v^2}{2\sigma_e^2\left(\sigma_e^2 + a_{i\cdot}\sigma_v^2\right)} \times M(1+\gamma), \text{ using } (13) \\
&\quad + \frac{1}{2\sigma_e^4} \times M\left(n_i\sigma_e^2 + a_{i\cdot}\sigma_v^2\right), \text{ using } (10) \\
&\quad - \frac{\sigma_v^2\left(2\sigma_e^2 + a_{i\cdot}\sigma_v^2\right)}{2\sigma_e^4\left(\sigma_e^2 + a_{i\cdot}\sigma_v^2\right)^2} \times M\left(a_{i\cdot}\sigma_e^2 + a_{i\cdot}^2\sigma_v^2\right), \text{ using } \mathbf{(14)} \\
&= -\frac{M\gamma\left[n_i\sigma_e^2 + (n_i-1)a_{i\cdot}\sigma_v^2\right]}{2\sigma_e^2\left(\sigma_e^2 + a_{i\cdot}\sigma_v^2\right)}
\end{aligned}
$$

C Matrix $\mathbf{J}^{(i)}$ at model

From the definition of $\mathbf{J}^{(i)}$, we get

$$
\begin{aligned}
\mathbf{J}_{\sigma_v^2}^{(i)} &= \int_{\mathbf{y}_i} \mathbf{u}_{\sigma_v^2}^2\left(\mathbf{y} \mid \mathbf{x}_i\right) f_{\boldsymbol{\theta}}^{1+\gamma}\left(\mathbf{y}_i \mid \mathbf{x}_i\right) d\mathbf{y}_i \\
&= \int_{\mathbf{y}_i} \left[-\frac{a_{i\cdot}}{2\left(\sigma_e^2 + a_{i\cdot}\sigma_v^2\right)} + \frac{1}{2\left(\sigma_e^2 + a_{i\cdot}\sigma_v^2\right)^2} \left\{\sum_{j=1}^{n_i} a_{ij}\left(y_{ij} - x_{ij}\boldsymbol{\beta}\right)\right\}^2 \right]^2 f_{\boldsymbol{\theta}}^{1+\gamma}\left(\mathbf{y}_i \mid \mathbf{x}_i\right) d\mathbf{y}_i \\
&= \frac{a_{i\cdot}^2}{4\left(\sigma_e^2 + a_{i\cdot}\sigma_v^2\right)^2} \int_{\mathbf{y}_i} f_{\boldsymbol{\theta}}^{1+\gamma}\left(\mathbf{y}_i \mid \mathbf{x}_i\right) d\mathbf{y}_i - \frac{a_{i\cdot}}{2\left(\sigma_e^2 + a_{i\cdot}\sigma_v^2\right)^3} \int_{\mathbf{y}_i} \left\{\sum_{j=1}^{n_i} a_{ij}\left(y_{ij} - x_{ij}\boldsymbol{\beta}\right)\right\}^2 f_{\boldsymbol{\theta}}^{1+\gamma} (\mathbf{y} \\
&\quad + \frac{1}{4\left(\sigma_e^2 + a_{i\cdot}\sigma_v^2\right)^4} \int_{\mathbf{y}_i} \left\{\sum_{j=1}^{n_i} a_{ij}\left(y_{it} - x_{it}\boldsymbol{\beta}\right)\right\}^4 f_{\boldsymbol{\theta}}^{1+\gamma}\left(\mathbf{y}_i \mid \mathbf{x}_i\right) d\mathbf{y}_i \\
&= \frac{a_{i\cdot}^2 M(1+\gamma)}{4\left(\sigma_e^2 + a_{i\cdot}\sigma_v^2\right)^2}, \text{ using } (13) \\
&\quad - \frac{a_{i\cdot}}{2\left(\sigma_e^2 + a_{i\cdot}\sigma_v^2\right)^3} \times M\left(a_{i\cdot}\sigma_e^2 + a_{i\cdot}^2\sigma_v^2\right) \text{ using } (14) \\
&\quad + \frac{1}{4\left(\sigma_e^2 + a_{i\cdot}\sigma_v^2\right)^4} \times \frac{3M\left(a_{i\cdot}\sigma_e^2 + a_{i\cdot}^2\sigma_v^2\right)^2}{1+\gamma} \text{using}(15) \\
&= \frac{M a_{i\cdot}^2\left(\gamma^2 + 2\right)}{4\left(\sigma_e^2 + a_{i\cdot}\sigma_v^2\right)^2(1+\gamma)}
\end{aligned}
$$

From Equations (??) and (??), we get

$$
\begin{aligned}
\mathbf{J}_{\boldsymbol{\beta}}^{(i)} =& \int_{\mathbf{y}_i} \mathbf{u}_{\boldsymbol{\beta}}\left(\mathbf{y}_i \mid \mathbf{x}_i\right) \mathbf{u}_{\boldsymbol{\beta}}^T\left(\mathbf{y}_i \mid \mathbf{x}_i\right) f_{\boldsymbol{\theta}}^{1+\gamma}\left(\mathbf{y}_i \mid \mathbf{x}_i\right) d\mathbf{y}_i \\
=& \int_{\mathbf{y}_i} \left[\frac{1}{\sigma_e^2} \sum_{j=1}^{n_i} a_{ij} x_{ij}\left(y_{ij}-x_{ij}\boldsymbol{\beta}\right) - \frac{a_{i\cdot}\sigma_v^2 \bar{\mathbf{x}}_i}{\sigma_e^2\left(\sigma_e^2+a_{i\cdot}\sigma_v^2\right)} \sum_{j=1}^{n_i} a_{ij}\left(y_{ij}-x_{ij}\boldsymbol{\beta}\right)\right] \\
& \left[\frac{1}{\sigma_e^2} \sum_{j=1}^{n_i} a_{ij} x_{ij}\left(y_{ij}-x_{ij}\boldsymbol{\beta}\right) - \frac{a_{i\cdot}\sigma_v^2 \bar{\mathbf{x}}_i}{\sigma_e^2\left(\sigma_e^2+a_{i\cdot}\sigma_v^2\right)} \sum_{j=1}^{n_i} a_{ij}\left(y_{ij}-x_{ij}\boldsymbol{\beta}\right)\right]^T f_{\boldsymbol{\theta}}^{1+\gamma}\left(\mathbf{y}_i \mid \mathbf{x}_i\right) d\mathbf{y}_i \\
=& \sum_{j=1}^{n_i} \left[\frac{1}{\sigma_e^4} x_{ij} x_{ij}^T - \frac{2a_{i\cdot}\sigma_v^2 x_{ij} \bar{\mathbf{x}}_i^T}{\sigma_e^4\left(\sigma_e^2+a_{i\cdot}\sigma_v^2\right)} + \frac{a_{i\cdot}^2\sigma_v^4 \bar{\mathbf{x}}_i \bar{\mathbf{x}}_i^T}{\sigma_e^4\left(\sigma_e^2+a_{i\cdot}\sigma_v^2\right)^2}\right] \int_{\mathbf{y}_i} a_{ij}^2\left(y_{ij}-x_{ij}\boldsymbol{\beta}\right)^2 f_{\boldsymbol{\theta}}^{1+\gamma}\left(\mathbf{y}_i \mid \mathbf{x}_i\right) d\mathbf{y} \\
& + \sum_{j\neq j'} \left[\frac{1}{\sigma_e^4} x_{ij} x_{ij'}^T - \frac{2a_{i\cdot}\sigma_v^2 x_{ij} \bar{\mathbf{x}}_i^T}{\sigma_e^4\left(\sigma_e^2+a_{i\cdot}\sigma_v^2\right)} + \frac{a_{i\cdot}^2\sigma_v^4 \bar{\mathbf{x}}_i \bar{\mathbf{x}}_i^T}{\sigma_e^4\left(\sigma_e^2+a_{i\cdot}\sigma_v^2\right)^2}\right] \int_{\mathbf{y}_i} a_{ij} a_{ij'}\left(y_{ij}-x_{ij}\boldsymbol{\beta}\right)\left(y_{ij'}-x_{ij'}\right. \\
=& M \sum_{j=1}^{n_i} \left[\frac{1}{\sigma_e^4} x_{ij} x_{ij}^T - \frac{2a_{i\cdot}\sigma_v^2 x_{ij} \bar{\mathbf{x}}_i^T}{\sigma_e^4\left(\sigma_e^2+a_{i\cdot}\sigma_v^2\right)} + \frac{a_{i\cdot}^2\sigma_v^4 \bar{\mathbf{x}}_i \bar{\mathbf{x}}_i^T}{\sigma_e^4\left(\sigma_e^2+a_{i\cdot}\sigma_v^2\right)^2}\right] a_{ij}\left(\sigma_e^2+a_{ij}\sigma_v^2\right), \ \textbf{using } (10) \\
& + M \sum_{j\neq j'} \left[\frac{1}{\sigma_e^4} x_{ij} x_{ij'}^T - \frac{2a_{i\cdot}\sigma_v^2 x_{ij} \bar{\mathbf{x}}_i^T}{\sigma_e^4\left(\sigma_e^2+a_{i\cdot}\sigma_v^2\right)} + \frac{a_{i\cdot}^2\sigma_v^4 \bar{\mathbf{x}}_i \bar{\mathbf{x}}_i^T}{\sigma_e^4\left(\sigma_e^2+a_{i\cdot}\sigma_v^2\right)^2}\right] a_{ij} a_{ij'} \sigma_v^2, \ \textbf{using } (11) \\
=& M\sigma_e^2 \sum_{j=1}^{n_i} \left[\frac{1}{\sigma_e^4} x_{ij} x_{ij}^T - \frac{2a_{i\cdot}\sigma_v^2 x_{ij} \bar{\mathbf{x}}_i^T}{\sigma_e^4\left(\sigma_e^2+a_{i\cdot}\sigma_v^2\right)} + \frac{a_{i\cdot}^2\sigma_v^4 \bar{\mathbf{x}}_i \bar{\mathbf{x}}_i^T}{\sigma_e^4\left(\sigma_e^2+a_{i\cdot}\sigma_v^2\right)^2}\right] a_{ij} \\
& + M\sigma_v^2 \sum_{j,j'=1}^{n_i} \left[\frac{1}{\sigma_e^4} x_{ij} x_{ij'}^T - \frac{2a_{i\cdot}\sigma_v^2 x_{ij} \bar{\mathbf{x}}_i^T}{\sigma_e^4\left(\sigma_e^2+a_{i\cdot}\sigma_v^2\right)} + \frac{a_{i\cdot}^2\sigma_v^4 \bar{\mathbf{x}}_i \bar{\mathbf{x}}_i^T}{\sigma_e^4\left(\sigma_e^2+a_{i\cdot}\sigma_v^2\right)^2}\right] a_{ij} a_{ij'} \\
=& M\sigma_e^2 \sum_{j=1}^{n_i} \frac{1}{\sigma_e^4} a_{ij} x_{ij} x_{ij}^T + M\sigma_e^2 \left[-\frac{2a_{i\cdot}^2\sigma_v^2 \bar{\mathbf{x}}_i \bar{\mathbf{x}}_i^T}{\sigma_e^4\left(\sigma_e^2+a_{i\cdot}\sigma_v^2\right)} + \frac{a_{i\cdot}^3\sigma_v^4 \bar{\mathbf{x}}_i \bar{\mathbf{x}}_i^T}{\sigma_e^4\left(\sigma_e^2+a_{i\cdot}\sigma_v^2\right)^2}\right] \\
& + M\sigma_v^2 \left[\frac{a_{i\cdot}^2}{\sigma_e^4} \bar{\mathbf{x}}_i \bar{\mathbf{x}}_i^T - \frac{2a_{i\cdot}^3\sigma_v^2 \bar{\mathbf{x}}_i \bar{\mathbf{x}}_i^T}{\sigma_e^4\left(\sigma_e^2+a_{i\cdot}\sigma_v^2\right)} + \frac{a_{i\cdot}^4\sigma_v^4 \bar{\mathbf{x}}_i \bar{\mathbf{x}}_i^T}{\sigma_e^4\left(\sigma_e^2+a_{i\cdot}\sigma_v^2\right)^2}\right] \\
=& M \left[\frac{\sigma_e^2}{\sigma_e^4} \sum_{j=1}^{n_i} a_{ij} x_{ij} x_{ij}^T + \frac{\sigma_v^2 a_{i\cdot}^2}{\sigma_e^4} \bar{\mathbf{x}}_i \bar{\mathbf{x}}_i^T + \left(\sigma_e^2+a_{i\cdot}\sigma_v^2\right)\left\{-\frac{2a_{i\cdot}^2\sigma_v^2 \bar{\mathbf{x}}_i \bar{\mathbf{x}}_i^T}{\sigma_e^4\left(\sigma_e^2+a_{i\cdot}\sigma_v^2\right)} + \frac{a_{i\cdot}^3\sigma_v^4 \bar{\mathbf{x}}_i \bar{\mathbf{x}}_i^T}{\sigma_e^4\left(\sigma_e^2+a_{i\cdot}\sigma_v^2\right)^2}\right\}\right] \\
=& M\sigma_e^{-4} \left[\sigma_e^2 \sum_{j=1}^{n_i} a_{ij} x_{ij} x_{ij}^T + \sigma_v^2 a_{i\cdot}^2 \bar{\mathbf{x}}_i \bar{\mathbf{x}}_i^T - 2\sigma_v^2 a_{i\cdot}^2 \bar{\mathbf{x}}_i \bar{\mathbf{x}}_i^T + \frac{a_{i\cdot}^3\sigma_v^4 \bar{\mathbf{x}}_i \bar{\mathbf{x}}_i^T}{\left(\sigma_e^2+a_{i\cdot}\sigma_v^2\right)}\right] \\
=& M\sigma_e^{-4} \left[\sigma_e^2 \sum_{j=1}^{n_i} a_{ij} x_{ij} x_{ij}^T + \sigma_v^2 a_{i\cdot}^2 \left(\frac{a_{i\cdot}\sigma_v^2}{\left(\sigma_e^2+a_{i\cdot}\sigma_v^2\right)} - 1\right) \bar{\mathbf{x}}_i \bar{\mathbf{x}}_i^T\right]
\end{aligned}
$$

$$
\begin{aligned}
\mathbf{J}_{\sigma_e^2}^{(i)} =& \int_{y_i} u_{\sigma_e^2}^2\left(y \mid \mathbf{x}_i\right) f_\theta^{1+\gamma}\left(y_i \mid \mathbf{x}_i\right) dy_i \\
=& \int_{y_i} \left[-\frac{n_i\sigma_e^2+(n_i-1)a_{i\cdot}\sigma_v^2}{2\sigma_e^2\left(\sigma_e^2+a_{i\cdot}\sigma_v^2\right)} + \frac{1}{2\sigma_e^4} \sum_{j=1}^{n_i} a_{ij}\left(y_{ij}-x_{ij}\beta\right)^2\right.
\end{aligned}
$$

$$
-\frac{\sigma_v^2\left(2\sigma_e^2+a_{i\cdot}\sigma_v^2\right)}{2\sigma_e^4\left(\sigma_e^2+a_{i\cdot}\sigma_v^2\right)^2}\left\{\sum_{j=1}^{n_i}a_{ij}\left(y_{it}-x_{it}\boldsymbol{\beta}\right)\right\}^2\Bigg]^2 f_\theta^{1+\gamma}\left(y_i\mid\mathbf{x}_i\right)dy_i
$$

$$
\begin{aligned}
=&\frac{\left(n_i\sigma_e^2+(n_i-1)a_{i\cdot}\sigma_v^2\right)^2}{4\sigma_e^4\left(\sigma_e^2+a_{i\cdot}\sigma_v^2\right)^2}\int_{y_i}f_\theta^{1+\gamma}\left(y_i\mid\mathbf{x}_i\right)dy_i\\
&+\frac{1}{4\sigma_e^8}\int_{y_i}\left\{\sum_{j=1}^{n_i}a_{ij}\left(y_{ij}-x_{ij}\beta\right)^2\right\}^2 f_\theta^{1+\gamma}\left(y_i\mid\mathbf{x}_i\right)dy_i\\
&+\frac{\sigma_v^4\left(2\sigma_e^2+a_{i\cdot}\sigma_v^2\right)^2}{4\sigma_e^8\left(\sigma_e^2+a_{i\cdot}\sigma_v^2\right)^4}\int_{y_i}\left\{\sum_{j=1}^{n_i}a_{ij}\left(y_{it}-x_{it}\boldsymbol{\beta}\right)\right\}^4 f_\theta^{1+\gamma}\left(y_i\mid\mathbf{x}_i\right)dy_i\\
&-\frac{n_i\sigma_e^2+(n_i-1)a_{i\cdot}\sigma_v^2}{2\sigma_e^6\left(\sigma_e^2+a_{i\cdot}\sigma_v^2\right)}\sum_{j=1}^{n_i}\int_{y_i}a_{ij}\left(y_{it}-x_{it}\beta\right)^2 f_\theta^{1+\gamma}\left(y_i\mid\mathbf{x}_i\right)dy_i\\
&+\frac{\left(n_i\sigma_e^2+(n_i-1)a_{i\cdot}\sigma_v^2\right)\sigma_v^2\left(2\sigma_e^2+a_{i\cdot}\sigma_v^2\right)}{2\sigma_e^6\left(\sigma_e^2+a_{i\cdot}\sigma_v^2\right)^3}\int_{y_i}\left\{\sum_{j=1}^{n_i}a_{ij}\left(y_{it}-x_{it}\boldsymbol{\beta}\right)\right\}^2 f_\theta^{1+\gamma}\left(y_i\mid\mathbf{x}_i\right)dy_i\\
&-\frac{\sigma_v^2\left(2\sigma_e^2+a_{i\cdot}\sigma_v^2\right)}{2\sigma_e^8\left(\sigma_e^2+a_{i\cdot}\sigma_v^2\right)^2}\sum_{j=1}^{n_i}\int_{y_i}a_{ij}\left(y_{ij}-x_{ij}\beta\right)^2\left\{\sum_{j=1}^{n_i}a_{ij}\left(y_{it}-x_{it}\boldsymbol{\beta}\right)\right\}^2 f_\theta^{1+\gamma}\left(y_i\mid\mathbf{x}_i\right)dy_i\\
=&\frac{\left(n_i\sigma_e^2+(n_i-1)a_{i\cdot}\sigma_v^2\right)^2}{4\sigma_e^4\left(\sigma_e^2+a_{i\cdot}\sigma_v^2\right)^2}\times M(1+\gamma),\textbf{using}(13).\\
&+\frac{1}{4\sigma_e^8}\frac{M\left\{\left(n_i^2+2n_i\right)\sigma_e^4+2(n_i+2)a_{i\cdot}\sigma_e^2\sigma_v^2+3a_{i\cdot}^2\sigma_v^4\right\}}{1+\gamma},\textbf{using}(16).\\
&+\frac{\sigma_v^4\left(2\sigma_e^2+a_{i\cdot}\sigma_v^2\right)^2}{4\sigma_e^8\left(\sigma_e^2+a_{i\cdot}\sigma_v^2\right)^4}\times\frac{3M\left(a_{i\cdot}\sigma_e^2+a_{i\cdot}^2\sigma_v^2\right)^2}{1+\gamma},\textbf{using}(15).\\
&-\frac{n_i\sigma_e^2+(n_i-1)a_{i\cdot}\sigma_v^2}{2\sigma_e^6\left(\sigma_e^2+a_{i\cdot}\sigma_v^2\right)}\times\sum_{j=1}^{n_i}M\left(\sigma_e^2+a_{ij}\sigma_v^2\right),\textbf{using}(10).\\
&+\frac{\left(n_i\sigma_e^2+(n_i-1)a_{i\cdot}\sigma_v^2\right)\sigma_v^2\left(2\sigma_e^2+a_{i\cdot}\sigma_v^2\right)}{2\sigma_e^6\left(\sigma_e^2+a_{i\cdot}\sigma_v^2\right)^3}\times M\left(a_{i\cdot}\sigma_e^2+a_{i\cdot}^2\sigma_v^2\right),\textbf{using}(14).\\
&-\frac{\sigma_v^2\left(2\sigma_e^2+a_{i\cdot}\sigma_v^2\right)}{2\sigma_e^8\left(\sigma_e^2+a_{i\cdot}\sigma_v^2\right)^2}\times M\left\{\frac{\left(n_ia_{i\cdot}+2a_{i\cdot}\right)\sigma_e^4+\left(n_ia_{i\cdot}^2+2a_{i\cdot}^2+3\sum_{j=1}^{n_i}a_{ij}^2\right)\sigma_e^2\sigma_v^2}{1+\gamma}\right.\\
&\qquad\left.+\frac{3\sigma_v^4\sum_{j=1}^{n_i}a_{ij}\left(a_{i\cdot}^2-a_{ij}a_{i\cdot}+a_{ij}^2\right)}{1+\gamma}\right\},\textbf{using}(17).\\
=&\frac{M(\gamma-1)\left(n_i\sigma_e^2+(n_i-1)a_{i\cdot}\sigma_v^2\right)^2}{4\sigma_e^4\left(\sigma_e^2+a_{i\cdot}\sigma_v^2\right)^2}\\
&+\frac{M\left\{\left(n_i^2+2n_i\right)\sigma_e^4+2(n_i+2)a_{i\cdot}\sigma_e^2\sigma_v^2+3a_{i\cdot}^2\sigma_v^4\right\}}{4\sigma_e^8(1+\gamma)}\\
&+\frac{3Ma_{i\cdot}^2\sigma_v^4\left(2\sigma_e^2+a_{i\cdot}\sigma_v^2\right)^2}{4(1+\gamma)\sigma_e^8\left(\sigma_e^2+a_{i\cdot}\sigma_v^2\right)^2}
\end{aligned}
$$

$$
-\frac{\sigma_v^2\left(2\sigma_e^2+a_{i\cdot}\sigma_v^2\right)}{2\sigma_e^8\left(\sigma_e^2+a_{i\cdot}\sigma_v^2\right)^2}\times M\left\{\frac{\left(n_i a_{i\cdot}+2a_{i\cdot}\right)\sigma_e^4+\left(n_i a_{i\cdot}^2+2a_{i\cdot}^2+3\sum_{j=1}^{n_i}a_{ij}^2\right)\sigma_e^2\sigma_v^2}{1+\gamma}\right.
$$

$$
\left.+\frac{3\sigma_v^4\sum_{j=1}^{n_i}a_{ij}\left(a_{i\cdot}^2-a_{ij}a_{i\cdot}+a_{ij}^2\right)}{1+\gamma}\right\}.
$$

From Equations (??) and (??), we get

$$
\begin{aligned}
\mathbf{J}_{\boldsymbol{\beta},\sigma_v^2}^{(i)}&=\int_{\mathbf{y}_i}\mathbf{u}_{\boldsymbol{\beta}}\left(\mathbf{y}_i\mid\mathbf{x}_i\right)\mathbf{u}_{\sigma_v^2}\left(\mathbf{y}_i\mid\mathbf{x}_i\right)f_{\boldsymbol{\theta}}^{1+\gamma}\left(\mathbf{y}_i\mid\mathbf{x}_i\right)d\mathbf{y}_i\\
&=\int_{\mathbf{y}_i}\left[\frac{1}{\sigma_e^2}\sum_{j=1}^{n_i}a_{ij}x_{ij}\left(y_{ij}-x_{ij}\boldsymbol{\beta}\right)-\frac{a_{i\cdot}\sigma_v^2\bar{\mathbf{x}}_i}{\sigma_e^2\left(\sigma_e^2+a_{i\cdot}\sigma_v^2\right)}\sum_{j=1}^{n_i}a_{ij}\left(y_{ij}-x_{ij}\boldsymbol{\beta}\right)\right]\\
&\quad\times\left[-\frac{a_{i\cdot}}{2\left(\sigma_e^2+a_{i\cdot}\sigma_v^2\right)}+\frac{1}{2\left(\sigma_e^2+a_{i\cdot}\sigma_v^2\right)^2}\left\{\sum_{j=1}^{n_i}a_{ij}\left(y_{ij}-x_{ij}\boldsymbol{\beta}\right)\right\}^2\right]f_{\boldsymbol{\theta}}^{1+\gamma}\left(\mathbf{y}_i\mid\mathbf{x}_i\right)d\mathbf{y}_i\\
&=0.\quad\textbf{as all odd moments similar to (12).}
\end{aligned}
$$

Similarly

$$
\begin{aligned}
\mathbf{J}_{\boldsymbol{\beta},\sigma_e^2}^{(i)}&=\int_{\mathbf{y}_i}\mathbf{u}_{\boldsymbol{\beta}}\left(\mathbf{y}_i\mid\mathbf{x}_i\right)\mathbf{u}_{\sigma_e^2}\left(\mathbf{y}_i\mid\mathbf{x}_i\right)f_{\theta}^{1+\gamma}\left(\mathbf{y}_i\mid\mathbf{x}_i\right)d\mathbf{y}_i\\
&=\int_{\mathbf{y}_i}\left[\frac{1}{\sigma_e^2}\sum_{j=1}^{n_i}a_{ij}x_{ij}\left(y_{ij}-x_{ij}\boldsymbol{\beta}\right)-\frac{a_{i\cdot}\sigma_v^2\bar{\mathbf{x}}_i}{\sigma_e^2\left(\sigma_e^2+a_{i\cdot}\sigma_v^2\right)}\sum_{j=1}^{n_i}a_{ij}\left(y_{ij}-x_{ij}\boldsymbol{\beta}\right)\right]\\
&\quad\times\left[-\frac{n_i\sigma_e^2+(n_i-1)a_{i\cdot}\sigma_v^2}{2\sigma_e^2\left(\sigma_e^2+a_{i\cdot}\sigma_v^2\right)}+\frac{1}{2\sigma_e^4}\sum_{j=1}^{n_i}a_{ij}\left(y_{ij}-x_{ij}\beta\right)^2\right.\\
&\quad\left.-\frac{\sigma_v^2\left(2\sigma_e^2+a_{i\cdot}\sigma_v^2\right)}{2\sigma_e^4\left(\sigma_e^2+a_{i\cdot}\sigma_v^2\right)^2}\left\{\sum_{j=1}^{n_i}a_{ij}\left(y_{it}-x_{it}\boldsymbol{\beta}\right)\right\}^2\right]f_{\boldsymbol{\theta}}^{1+\gamma}\left(\mathbf{y}_i\mid\mathbf{x}_i\right)d\mathbf{y}_i\\
&=0.
\end{aligned}
$$

$$
\begin{aligned}
\mathbf{J}_{\sigma_v^2,\sigma_e^2}^{(i)}&=\int_{\mathbf{y}_i}\mathbf{u}_{\sigma_v^2}\left(\mathbf{y}_i\mid\mathbf{x}_i\right)\mathbf{u}_{\sigma_e^2}\left(\mathbf{y}_i\mid\mathbf{x}_i\right)f_{\boldsymbol{\theta}}^{1+\gamma}\left(\mathbf{y}_i\mid\mathbf{x}_i\right)d\mathbf{y}_i\\
&=\int_{\mathbf{y}_i}\left[-\frac{a_{i\cdot}}{2\left(\sigma_e^2+a_{i\cdot}\sigma_v^2\right)}+\frac{1}{2\left(\sigma_e^2+a_{i\cdot}\sigma_v^2\right)^2}\left\{\sum_{j=1}^{n_i}a_{ij}\left(y_{ij}-x_{ij}\boldsymbol{\beta}\right)\right\}^2\right]\\
&\quad\times\left[-\frac{n_i\sigma_e^2+(n_i-1)a_{i\cdot}\sigma_v^2}{2\sigma_e^2\left(\sigma_e^2+a_{i\cdot}\sigma_v^2\right)}+\frac{1}{2\sigma_e^4}\sum_{j=1}^{n_i}a_{ij}\left(y_{ij}-x_{ij}\beta\right)^2\right.\\
&\quad\left.-\frac{\sigma_v^2\left(2\sigma_e^2+a_{i\cdot}\sigma_v^2\right)}{2\sigma_e^4\left(\sigma_e^2+a_{i\cdot}\sigma_v^2\right)^2}\left\{\sum_{j=1}^{n_i}a_{ij}\left(y_{it}-x_{it}\boldsymbol{\beta}\right)\right\}^2\right]f_{\boldsymbol{\theta}}^{1+\gamma}\left(\mathbf{y}_i\mid\mathbf{x}_i\right)d\mathbf{y}_i\\
&=\frac{n_i a_{i\cdot}\sigma_e^2+(n_i-1)a_{i\cdot}^2\sigma_v^2}{4\sigma_e^2\left(\sigma_e^2+a_{i\cdot}\sigma_v^2\right)^2}\int_{\mathbf{y}_i}f_{\boldsymbol{\theta}}^{1+\gamma}\left(\mathbf{y}_i\mid\mathbf{x}_i\right)d\mathbf{y}_i
\end{aligned}
$$

$$
\begin{aligned}
&+\frac{1}{4\sigma_e^2\left(\sigma_e^2+a_{i\cdot}\sigma_v^2\right)^2}\sum_{j=1}^{n_i}\int_{\mathbf{y}_i} a_{ij}\left(y_{ij}-x_{ij}\beta\right)^2\left\{\sum_{j=1}^{n_i} a_{ij}\left(y_{ij}-x_{ij}\boldsymbol{\beta}\right)\right\}^2 f_{\boldsymbol{\theta}}^{1+\gamma}\left(\mathbf{y}_i \mid \mathbf{x}_i\right) d\mathbf{y}_i \\
&-\frac{\sigma_v^2\left(2\sigma_e^2+a_{i\cdot}\sigma_v^2\right)}{4\sigma_e^2\left(\sigma_e^2+a_{i\cdot}\sigma_v^2\right)^4}\int_{\mathbf{y}_i}\left\{\sum_{j=1}^{n_i} a_{ij}\left(y_{ij}-x_{ij}\boldsymbol{\beta}\right)\right\}^4 f_{\boldsymbol{\theta}}^{1+\gamma}\left(\mathbf{y}_i \mid \mathbf{x}_i\right) d\mathbf{y}_i \\
&-\frac{a_{i\cdot}}{4\sigma_e^4\left(\sigma_e^2+a_{i\cdot}\sigma_v^2\right)}\int_{\mathbf{y}_i}\sum_{j=1}^{n_i} a_{ij}\left(y_{ij}-x_{ij}\beta\right)^2 f_{\boldsymbol{\theta}}^{1+\gamma}\left(\mathbf{y}_i \mid \mathbf{x}_i\right) d\mathbf{y}_i \\
&+\left[\frac{a_{i\cdot}\sigma_v^2\left(2\sigma_e^2+a_{i\cdot}\sigma_v^2\right)}{4\sigma_e^4\left(\sigma_e^2+a_{i\cdot}\sigma_v^2\right)^3}-\frac{n_i\sigma_e^2+(n_i-1)a_{i\cdot}\sigma_v^2}{4\sigma_e^2\left(\sigma_e^2+a_{i\cdot}\sigma_v^2\right)^3}\right]\int_{\mathbf{y}_i}\left\{\sum_{j=1}^{n_i} a_{ij}\left(y_{ij}-x_{ij}\boldsymbol{\beta}\right)\right\}^2 f_{\boldsymbol{\theta}}^{1+\gamma}\left(\mathbf{y}_i \mid \mathbf{x}_i\right. \text{[illegible]} \\
=&\frac{n_i a_{i\cdot}\sigma_e^2+(n_i-1)a_{i\cdot}^2\sigma_v^2}{4\sigma_e^2\left(\sigma_e^2+a_{i\cdot}\sigma_v^2\right)^2}\times M(1+\gamma), \textbf{using}(13) \\
&+\frac{1}{4\sigma_e^2\left(\sigma_e^2+a_{i\cdot}\sigma_v^2\right)^2}\times M\left\{\frac{\left(n_i a_{i\cdot}+2a_{i\cdot}\right)\sigma_e^4+\left(n_i a_{i\cdot}^2+2a_{i\cdot}^2+3\sum_{j=1}^{n_i} a_{ij}^2\right)\sigma_e^2\sigma_v^2}{1+\gamma}\right. \\
&\qquad\qquad\left.+\frac{3\sigma_v^4\sum_{j=1}^{n_i} a_{ij}\left(a_{i\cdot}^2-a_{ij}a_{i\cdot}+a_{ij}^2\right)}{1+\gamma}\right\}, \textbf{using}(17). \\
&-\frac{\sigma_v^2\left(2\sigma_e^2+a_{i\cdot}\sigma_v^2\right)}{4\sigma_e^2\left(\sigma_e^2+a_{i\cdot}\sigma_v^2\right)^4}\times\frac{3M\left(a_{i\cdot}\sigma_e^2+a_{i\cdot}^2\sigma_v^2\right)^2}{1+\gamma}, \textbf{using}(15). \\
&-\frac{a_{i\cdot}}{4\sigma_e^4\left(\sigma_e^2+a_{i\cdot}\sigma_v^2\right)}\times M\left(n_i\sigma_e^2+a_{i\cdot}\sigma_v^2\right), \textbf{using}(10). \\
&+\left[\frac{a_{i\cdot}\sigma_v^2\left(2\sigma_e^2+a_{i\cdot}\sigma_v^2\right)}{4\sigma_e^4\left(\sigma_e^2+a_{i\cdot}\sigma_v^2\right)^3}-\frac{n_i\sigma_e^2+(n_i-1)a_{i\cdot}\sigma_v^2}{4\sigma_e^2\left(\sigma_e^2+a_{i\cdot}\sigma_v^2\right)^3}\right]\times M\left(a_{i\cdot}\sigma_e^2+a_{i\cdot}^2\sigma_v^2\right), \textbf{using}(14). \\
=&M(1+\gamma)\frac{n_i a_{i\cdot}\sigma_e^2+(n_i-1)a_{i\cdot}^2\sigma_v^2}{4\sigma_e^2\left(\sigma_e^2+a_{i\cdot}\sigma_v^2\right)^2} \\
&+\frac{M}{4\sigma_e^2\left(\sigma_e^2+a_{i\cdot}\sigma_v^2\right)^2}\left\{\frac{\left(n_i a_{i\cdot}+2a_{i\cdot}\right)\sigma_e^4+\left(n_i a_{i\cdot}^2-4a_{i\cdot}^2+3\sum_{j=1}^{n_i} a_{ij}^2\right)\sigma_e^2\sigma_v^2}{1+\gamma}\right. \\
&\qquad\qquad\left.+\frac{3\sigma_v^4\sum_{j=1}^{n_i} a_{ij}\left(-a_{ij}a_{i\cdot}+a_{ij}^2\right)}{1+\gamma}\right\} \\
&-\frac{M a_{i\cdot}\left(n_i\sigma_e^2-(n_i-1)a_i\sigma_v^2\right)}{2\sigma_e^2\left(\sigma_e^2+a_{i\cdot}\sigma_v^2\right)^2}
\end{aligned}
$$

D Integrals for $\mathbf{J}^{(i)}$

$$
\begin{aligned}
&\int_{\mathbf{y}_i} a_{ij}a_{ij'}\left(y_{ij}-x_{ij}\boldsymbol{\beta}\right)\left(y_{ij'}-x_{ij'}\boldsymbol{\beta}\right) f_{\boldsymbol{\theta}}^{1+\gamma}\left(\mathbf{y}_i \mid \mathbf{x}_i\right) d\mathbf{y}_i \\
&=\int_{z_i} a_{ij}a_{ij'}z_{ij}z_{ij'}f_{\boldsymbol{\theta}}^{1+\gamma}\left(z_i \mid 0\right) dz_i\textbf{, where } f_{\boldsymbol{\theta}}\left(z_i \mid 0\right) \textbf{ is } N_{n_i}(0,\mathbf{V}_i) \\
&=(2\pi)^{-\frac{n_i\gamma}{2}}|\mathbf{V}_i|^{-\frac{\gamma}{2}}\int_{z_i} a_{ij}a_{ij'}z_{ij}z_{ij'}(2\pi)^{-\frac{n_i}{2}}|\mathbf{V}_i|^{-\frac{1}{2}}\exp\left\{-\frac{1+\gamma}{2}z_i'\mathbf{V}_i^{-1}z_i\right\} dz_i
\end{aligned}
$$

$$
\begin{aligned}
&= (2\pi)^{-\frac{n_i\gamma}{2}}|\mathbf{V}_i|^{-\frac{\gamma}{2}}(1+\gamma)^{-\frac{n_i}{2}}\int_{z_i} a_{ij}a_{ij'}z_{ij}z_{ij'}(2\pi)^{-\frac{n_i}{2}}\left|\frac{\mathbf{V}_i}{1+\gamma}\right|^{-\frac{1}{2}}\exp\left\{-\frac{1}{2}z_i'\left(\frac{\mathbf{V}_i}{1+\gamma}\right)^{-1}z_i\right\}dz_{[illegible]}\\
&= (2\pi)^{-\frac{n_i\gamma}{2}}|\mathbf{V}_i|^{-\frac{\gamma}{2}}(1+\gamma)^{-\frac{n_i+2}{2}}a_{ij}a_{ij'}\mathbf{V}_{i-jj'}\\
&= Ma_{ij}a_{ij'}\mathbf{V}_{i-jj'}. \qquad (8)
\end{aligned}
$$

where

$$
\begin{aligned}
M &= (2\pi)^{-\frac{n_i\gamma}{2}}|\mathbf{V}_i|^{-\frac{\gamma}{2}}(1+\gamma)^{-\frac{n_i+2}{2}}\\
&= (2\pi)^{-\frac{n_i\gamma}{2}}(1+\gamma)^{-\frac{n_i+2}{2}}\left(\sigma_e^{2(n_i-1)}(\sigma_e^2+a_{i\cdot}\sigma_v^2)\prod_{j=1}^{n_i}k_{ij}^2\right)^{-\frac{\gamma}{2}}\\
&= (2\pi)^{-\frac{n_i\gamma}{2}}(1+\gamma)^{-\frac{n_i+2}{2}}\sigma_e^{2(-\frac{\gamma(n_i-1)}{2})}(\sigma_e^2+a_{i\cdot}\sigma_v^2)^{-\frac{\gamma}{2}}\prod_{j=1}^{n_i}k_{ij}^{-\gamma}. \qquad (9)
\end{aligned}
$$

When $j=j'$, the (8) can be writen as

$$
\int_{\mathbf{y}_i} a_{ij}^2\left(y_{ij}-x_{ij}\boldsymbol{\beta}\right)^2 f_{\boldsymbol{\theta}}^{1+\gamma}\left(\mathbf{y}_i \mid \mathbf{x}_i\right)d\mathbf{y}_i = Ma_{ij}\left(\sigma_e^2+a_{ij}\sigma_v^2\right). \qquad (10)
$$

For $j \neq j'$, combining (8), we get

$$
\int_{\mathbf{y}_i} a_{ij}a_{ij'}\left(y_{ij}-x_{ij}\boldsymbol{\beta}\right)\left(y_{ij'}-x_{ij'}\boldsymbol{\beta}\right)f_{\boldsymbol{\theta}}^{1+\gamma}\left(\mathbf{y}_i \mid \mathbf{x}_i\right)d\mathbf{y}_i = Ma_{ij}a_{ij'}\sigma_v^2. \qquad (11)
$$

For two integer r and s, where $(r+s)$ is an odd number, we have

$$
\begin{aligned}
&\int_{\mathbf{y}_i} a_{ij}^r a_{ij'}^s\left(y_{ij}-x_{ij}\boldsymbol{\beta}\right)^r\left(y_{ij}-x_{ij}\boldsymbol{\beta}\right)^s f_{\boldsymbol{\theta}}^{1+\gamma}\left(\mathbf{y}_i \mid \mathbf{x}_i\right)d\mathbf{y}_i\\
&= \int_{z_i} a_{ij}^r a_{ij'}^s z_{ij}^r z_{ij}^s f_{\boldsymbol{\theta}}^{1+\gamma}\left(z_i \mid 0\right)dz_i, \ \textbf{where } f_{\boldsymbol{\theta}}\left(z_i \mid 0\right) \ \textbf{is } N_T(0,\mathbf{V}_i)\\
&= 0, \ \textbf{using } (22). \qquad (12)
\end{aligned}
$$

Now

$$
\begin{aligned}
\int_{\mathbf{y}_i} f_{\boldsymbol{\theta}}^{1+\gamma}\left(\mathbf{y}_i \mid \mathbf{x}_i\right)d\mathbf{y}_i &= \int_{z_i} f_{\boldsymbol{\theta}}^{1+\gamma}\left(z_i \mid 0\right)dz_i, \textbf{ where } f_{\boldsymbol{\theta}}\left(z_i \mid 0\right) \ \textbf{is } N_T(0,\mathbf{V}_i)\\
&= (2\pi)^{-\frac{n_i\gamma}{2}}|\mathbf{V}_i|^{-\frac{\gamma}{2}}\int_{z_i}(2\pi)^{-\frac{n_i}{2}}|\mathbf{V}_i|^{-\frac{1}{2}}\exp\left\{-\frac{1+\gamma}{2}z_i'\mathbf{V}_i^{-1}z_i\right\}dz_i\\
&= (2\pi)^{-\frac{n_i\gamma}{2}}|\mathbf{V}_i|^{-\frac{\gamma}{2}}(1+\gamma)^{-\frac{n_i}{2}}\int_{z_i}(2\pi)^{-\frac{n_i}{2}}\left|\frac{\mathbf{V}_i}{1+\gamma}\right|^{-\frac{1}{2}}\exp\left\{-\frac{1}{2}z_i'\left(\frac{\mathbf{V}_i}{1+\gamma}\right)^{-[illegible]}\right.\\
&= (2\pi)^{-\frac{n_i\gamma}{2}}|\mathbf{V}_i|^{-\frac{\gamma}{2}}(1+\gamma)^{-\frac{n_i}{2}}\\
&= M(1+\gamma), \qquad (13)
\end{aligned}
$$

So

$$
\int_{\mathbf{y}_i}\left\{\sum_{j=1}^{n_i}a_{ij}\left(y_{ij}-x_{ij}\boldsymbol{\beta}\right)\right\}^2 f_{\boldsymbol{\theta}}^{1+\gamma}\left(\mathbf{y}_i \mid \mathbf{x}_i\right)d\mathbf{y}_i
$$

$$= \int_{z_i} \left\{ \sum_{j=1}^{n_i} a_{ij} z_{ij} \right\}^2 f_{\boldsymbol{\theta}}^{1+\gamma}(z_i \mid 0)\, dz_i\textbf{, where } f_{\boldsymbol{\theta}}(z_i \mid 0) \textbf{ is } N_{n_i}(0, \mathbf{V}_i)$$

$$= (2\pi)^{-\frac{n_i\gamma}{2}} |\mathbf{V}_i|^{-\frac{\gamma}{2}} \int_{z_i} \left\{ \sum_{j=1}^{n_i} a_{ij} z_{ij} \right\}^2 (2\pi)^{-\frac{n_i}{2}} |\mathbf{V}_i|^{-\frac{1}{2}} \exp\left\{ -\frac{1+\gamma}{2} z_i' \mathbf{V}_i^{-1} z_i \right\} dz_i$$

$$= (2\pi)^{-\frac{n_i\gamma}{2}} |\mathbf{V}_i|^{-\frac{\gamma}{2}} (1+\gamma)^{-\frac{n_i}{2}} \int_{z_i} \left\{ \sum_{j=1}^{n_i} a_{ij} z_{ij} \right\}^2 (2\pi)^{-\frac{n_i}{2}} \left| \frac{\mathbf{V}_i}{1+\gamma} \right|^{-\frac{1}{2}} \exp\left\{ -\frac{1}{2} z_i' \left(\frac{\mathbf{V}_i}{1+\gamma} \right)^{-1} z \right.$$

$$= M(1+\gamma)\, \mathrm{E}\left(\left[\sum_{j=1}^{n_i} a_{ij} s_{ij} \right]^2 \right), \textbf{ using } (9)$$

$$= M\left(a_{i\cdot}\sigma_e^2 + a_{i\cdot}^2 \sigma_v^2 \right), \textbf{using}(18). \tag{14}$$

Similarly

$$\int_{\mathbf{y}_i} \left\{ \sum_{j=1}^{n_i} a_{ij} (y_{ij} - x_{ij}\boldsymbol{\beta}) \right\}^4 f_{\boldsymbol{\theta}}^{1+\gamma}(\mathbf{y}_i \mid \mathbf{x}_i)\, d\mathbf{y}_i = M(1+\gamma)\, \mathrm{E}\left(\left[\sum_{j=1}^{n_i} a_{ij} s_{ij} \right]^4 \right)$$

$$= \frac{3M\left(a_{i\cdot}\sigma_e^2 + a_{i\cdot}^2 \sigma_v^2 \right)^2}{1+\gamma}. \tag{15}$$

$$\int_{\mathbf{y}_i} \left\{ \sum_{j=1}^{n_i} a_{ij} (y_{ij} - x_{ij}\boldsymbol{\beta})^2 \right\}^2 f_{\boldsymbol{\theta}}^{1+\gamma}(\mathbf{y}_i \mid \mathbf{x}_i)\, d\mathbf{y}_i = \int_{\mathbf{y}_i} \left\{ \sum_{j=1}^{n_i} a_{ij} z_{ij}^2 \right\}^2 f_{\boldsymbol{\theta}}^{1+\gamma}(z_i \mid 0)\, dz_i$$

$$= M(1+\gamma)\, \mathrm{E}\left(\left[\sum_{j=1}^{n_i} a_{ij} s_{ij}^2 \right]^2 \right)$$

$$= \frac{M\left\{ \left(n_i^2 + 2n_i \right)\sigma_e^4 + 2(n_i+2) a_{i\cdot}\sigma_e^2\sigma_v^2 + 3a_{i\cdot}^2\sigma_v^4 \right\}}{1+\gamma}, \textbf{ using (20).} \tag{16}$$

and

$$\int_{\mathbf{y}_i} a_{ij'} (y_{ij'} - x_{ij'}\boldsymbol{\beta})^2 \left\{ \sum_{j=1}^{n_i} a_{ij} (y_{ij} - x_{ij}\boldsymbol{\beta}) \right\}^2 f_{\boldsymbol{\theta}}^{1+\gamma}(\mathbf{y}_i \mid \mathbf{x}_i)\, d\mathbf{y}_i$$

$$= M(1+\gamma)\, \mathrm{E}\left(a_{ij'} s_{ij'}^2 \left[\sum_{j=1}^{n_i} a_{ij} s_{ij} \right]^2 \right)$$

$$= \frac{M\left\{ (a_{i\cdot} + 2a_{ij'})\sigma_e^4 + \left(a_{i\cdot}^2 + 2a_{ij'}a_{i\cdot} + 4a_{ij}^2 - a_{ij'}^2 \right)\sigma_e^2\sigma_v^2 + 3a_{ij'}\left(a_{i\cdot}^2 - a_{ij'}a_{i\cdot} + a_{ij'}^2 \right)\sigma_v^4 \right\}}{1+\gamma}. \textbf{ u} \tag{17}$$

E Expectations for Integrals

Suppose $s_i \sim N_{n_i}\left(0, \frac{\boldsymbol{V}_i}{1+\gamma}\right)$**, then**

$$
\begin{aligned}
\mathrm{V}\left(\sum_{j=1}^{n_i} a_{ij}s_{ij}\right) &= \sum_{j=1}^{n_i}\mathrm{V}\left(a_{ij}s_{ij}\right) + \sum_{j\neq j'}\mathrm{cov}\left(a_{ij}a_{ij'}s_{ij}s_{ij'}\right)\\
&= \frac{1}{1+\gamma}\sum_{j=1}^{n_i} a_{ij}^2 \boldsymbol{V}_{i-jj} + \frac{1}{1+\gamma}\sum_{j\neq j'} a_{ij}a_{ij'}\boldsymbol{V}_{i-jj'}\\
&= \frac{1}{1+\gamma}\sum_{j=1}^{n_i} a_{ij}^2\left(k_{ij}^2\sigma_e^2+\sigma_v^2\right) + \frac{1}{1+\gamma}\sum_{j\neq j'} a_{ij}a_{ij'}\sigma_v^2\\
&= \frac{1}{1+\gamma}\left(\sigma_e^2\sum_{j=1}^{n_i} a_{ij} + \sigma_v^2\sum_{j=1}^{n_i} a_{ij}^2\right) + \frac{1}{1+\gamma}\sigma_v^2\sum_{j\neq j'} a_{ij}a_{ij'}\\
&= \frac{1}{1+\gamma}\left(a_{i\cdot}\sigma_e^2 + a_{i\cdot}^2\sigma_v^2\right),
\end{aligned}
$$

where $\boldsymbol{V}_{i-jj'}$ is the element of $\mathbf{V}_i$ in j row and j' column.

So $\sum_{j=1}^{n_i} a_{ij}s_{ij} \sim N\left(0, \frac{1}{1+\gamma}\left(a_{i\cdot}\sigma_e^2 + a_i^T a_i \sigma_v^2\right)\right)$. Therefore

$$
\mathrm{E}\left(\left[\sum_{j=1}^{n_i} a_{ij}s_{ij}\right]^2\right) = \frac{1}{1+\gamma}\left(a_{i\cdot}\sigma_e^2 + a_{i\cdot}^2\sigma_v^2\right), \tag{18}
$$

$$
\mathrm{E}\left(\left[\sum_{j=1}^{n_i} a_{ij}s_{ij}\right]^4\right) = \frac{3}{(1+\gamma)^2}\left(a_{i\cdot}\sigma_e^2 + a_{i\cdot}^2\sigma_v^2\right)^2. \tag{19}
$$

and

$$
\begin{aligned}
&\mathrm{E}\left(\left[\sum_{j=1}^{n_i} a_{ij}s_{ij}^2\right]^2\right) = \mathrm{E}\left(\sum_{j=1}^{n_i} a_{ij}^2 s_{ij}^4 + \sum_{j\neq j'} a_{ij}a_{ij'}s_{ij}^2 s_{ij'}^2\right)\\
&= \sum_{j=1}^{n_i}\frac{3a_{ij}^2\boldsymbol{V}_{i-jj}^2}{(1+\gamma)^2} + \sum_{j\neq j'}\frac{a_{ij}a_{ij'}\left(\boldsymbol{V}_{i-jj}\boldsymbol{V}_{i-j'j'} + 2\boldsymbol{V}_{i-jj'}^2\right)}{(1+\gamma)^2}\\
&= \frac{3\sum_{j=1}^{n_i} a_{ij}^2\left(k_{ij}^2\sigma_e^2+\sigma_v^2\right)^2}{(1+\gamma)^2} + \frac{\sum_{j\neq j'} a_{ij}a_{ij'}\left(k_{ij}^2\sigma_e^2+\sigma_v^2\right)\left(k_{ij'}^2\sigma_e^2+\sigma_v^2\right) + 2\sum_{j\neq j'} a_{ij}a_{ij'}\sigma_v^4}{(1+\gamma)^2}\\
&= \frac{2\sum_{j=1}^{n_i}\left(\sigma_e^2+a_{ij}\sigma_v^2\right)^2 + \left(\sum_{j=1}^{n_i}\left(\sigma_e^2+a_{ij}\sigma_v^2\right)\right)^2 + 2\sum_{j\neq j'} a_{ij}a_{ij'}\sigma_v^4}{(1+\gamma)^2}\\
&= \frac{2n_i\sigma_e^4 + 4\sigma_e^2\sigma_v^2\sum_{j=1}^{n_i} a_{ij} + 2\sigma_v^4\sum_{j=1}^{n_i} a_{ij}^2 + n_i^2\sigma_e^4 + 2n_i a_{i\cdot}\sigma_e^2\sigma_v^2 + a_{i\cdot}^2\sigma_v^4 + 2\sigma_v^4\sum_{j\neq j'} a_{ij}a_{ij'}}{(1+\gamma)^2}\\
&= \frac{\left(n_i^2+2n_i\right)\sigma_e^4 + 2(n_i+2)a_{i\cdot}\sigma_e^2\sigma_v^2 + 3a_{i\cdot}^2\sigma_v^4}{(1+\gamma)^2}.
\end{aligned} \tag{20}
$$

For $j' = 1, 2, \cdots, n_i$, we have

$$
\mathrm{E}\left(a_{ij'}s_{ij'}^2\left[\sum_{j=1}^{n_i} a_{ij}s_{ij}\right]^2\right)
$$

$$= \mathrm{E}\left(a_{ij'}^3 s_{ij'}^4 + a_{ij'} s_{ij'}^2 \sum_{j\neq j'} a_{ij}^2 s_{ij}^2 + a_{ij'}^2 s_{ij'}^3 \sum_{j\neq j'} a_{ij} s_{ij} + a_{ij'} s_{ij'}^2 \sum_{j\neq j'\neq j''} a_{ij} a_{ij''} s_{ij} s_{ij''} \right)$$

$$= \left\{ 3a_{ij'}^3 \boldsymbol{V}_{i-j'j'}^2 + \left(a_{ij'} \boldsymbol{V}_{i-j'j'} \sum_{j\neq j'} a_{ij}^2 \boldsymbol{V}_{i-jj} + 2a_{ij'} \sum_{j\neq j'} a_{ij}^2 \boldsymbol{V}_{i-jj'}^2 \right) + 3a_{ij'}^2 \boldsymbol{V}_{i-j'j'} \sum_{j\neq j'} a_{ij} \boldsymbol{V}_{i-} \text{[illegible]} \right.$$

$$\left. \left(a_{ij'} \boldsymbol{V}_{i-j'j'} \sum_{j\neq j'\neq j''} a_{ij} a_{ij''} \boldsymbol{V}_{i-jj''} + 2a_{ij'} \sum_{j\neq j'\neq j''} a_{ij} a_{ij''} \boldsymbol{V}_{i-jj'} \boldsymbol{V}_{i-j'j''} \right) \right\} \frac{1}{(1+\gamma)^2}$$

$$= \left\{ 2a_{ij'}^3 \left(k_{ij'}^2 \sigma_e^2 + \sigma_v^2\right)^2 + a_{ij'} \left(k_{ij'}^2 \sigma_e^2 + \sigma_v^2\right) \sum_{j=1}^{n_i} a_{ij}^2 \left(k_{ij}^2 \sigma_e^2 + \sigma_v^2\right) + 2a_{ij'} \sum_{j\neq j'} a_{ij}^2 \sigma_v^4 \right.$$

$$\left. + 3a_{ij'}^2 \left(k_{ij'}^2 \sigma_e^2 + \sigma_v^2\right) \sum_{j\neq j'} a_{ij} \sigma_v^2 + a_{ij'} \left(k_{ij'}^2 \sigma_e^2 + \sigma_v^2\right) \sum_{j\neq j'\neq j''} a_{ij} a_{ij''} \sigma_v^2 + 2a_{ij'} \sum_{j\neq j'\neq j''} a_{ij} a_{ij''} \sigma_v^4 \right\} \text{[illegible]}$$

$$= \left\{ 2a_{ij'} \left(\sigma_e^2 + a_{ij'} \sigma_v^2\right)^2 + \left(\sigma_e^2 + a_{ij'} \sigma_v^2\right) \left(a_{i\cdot} \sigma_e^2 + \sigma_v^2 \sum_{j=1}^{n_i} a_{ij}^2 \right) + 2a_{ij'} \sum_{j\neq j'} a_{ij}^2 \sigma_v^4 \right.$$

$$\left. + 3a_{ij'} \left(\sigma_e^2 + a_{ij'} \sigma_v^2\right) \sum_{j\neq j'} a_{ij} \sigma_v^2 + \left(\sigma_e^2 + a_{ij'} \sigma_v^2\right) \sum_{j\neq j'\neq j''} a_{ij} a_{ij''} \sigma_v^2 + 2a_{ij'} \sum_{j\neq j'\neq j''} a_{ij} a_{ij''} \sigma_v^4 \right\} \text{(1 [illegible]}$$

$$= \left\{ \left(a_{i\cdot} + 2a_{ij'}\right) \sigma_e^4 + \left(4a_{ij}^2 + \sum_{j=1}^{n_i} a_{ij}^2 + a_{ij'} a_{i\cdot} + 3a_{ij'} \sum_{j\neq j'} a_{ij} + \sum_{j\neq j'\neq j''} a_{ij} a_{ij''} \right) \sigma_e^2 \sigma_v^2 \right.$$

$$\left. + \left(2a_{ij'}^3 + 2a_{ij'} \sum_{j\neq j'} a_{ij}^2 + a_{ij'} \sum_{j=1}^{n_i} a_{ij}^2 + 3a_{ij'}^2 \sum_{j\neq j'} a_{ij} + 3a_{ij'} \sum_{j\neq j'\neq j''} a_{ij} a_{ij''} \right) \sigma_v^4 \right\} \frac{1}{(1+\gamma)^2}$$

$$= \left\{ \left(a_{i\cdot} + 2a_{ij'}\right) \sigma_e^4 + \left(a_{i\cdot}^2 + 2a_{ij'} a_{i\cdot} + 4a_{ij}^2 - a_{ij'}^2 \right) \sigma_e^2 \sigma_v^2 + 3a_{ij'} \left(a_{i\cdot}^2 - a_{ij'} a_{i\cdot} + a_{ij'}^2 \right) \sigma_v^4 \right\} \frac{1}{(1+\gamma)^2} \text{[illegible]} \quad (21)$$

where the formula

$$\sum_{j\neq j'\neq j''} a_{ij} a_{ij''} = \sum_{j,j''} a_{ij} a_{ij''} - \sum_{j=j''\neq j'} a_{ij}^2 - \sum_{j=j'\neq j''} a_{ij'} a_{ij''} - \sum_{j''=j'\neq j} a_{ij'} a_{ij} - a_{ij'}^2$$
$$= a_{i\cdot}^2 - \sum_{j\neq j'} a_{ij}^2 - 2\sum_{j\neq j'} a_{ij'} a_{ij} - a_{ij'}^2$$
$$= a_{i\cdot}^2 - \sum_{j=1}^{n_i} a_{ij}^2 - 2a_{ij'} \sum_{j\neq j'} a_{ij}.$$

was used in above.
Similarly

$$\mathrm{E}\left(a_{ij'} s_{ij'} \left[\sum_{j=1}^{n_i} a_{ij} s_{ij} \right]^2 \right) = \mathrm{E}\left(a_{ij'}^3 s_{ij'}^3 + a_{ij'}^2 s_{ij'}^2 \sum_{j\neq j'} a_{ij} s_{ij} + a_{ij'} s_{ij'} \sum_{j\neq j'\neq j''} a_{ij} a_{ij''} s_{ij} s_{ij''} \right) = \text{[illegible]} \quad (22)$$

Acknowledgement

We are grateful Lanzhou University of Finance and Economics for providing with a learning and research platform.

References

1. Marshall RJ. Mapping disease and mortality rates using empirical Bayes estimators. Journal of the Royal Statistical Society: Series C (Applied Statistics). 1991; 40(2):283-294.
2. Clayton D, Kaldor J. Empirical Bayes estimates of age-standardized relative risks for use in disease mapping. Biometrics. 1987; p.671-681.
3. Battese GE, Harter RM, Fuller WA. An error-components model for prediction of county crop areas using survey and satellite data. Journal of the American Statistical Association. 1988; 83(401):28-36.
4. Cruze NB, Erciulescu AL, Nandram B, et al. Producing official county-level agricultural estimates in the United States: Needs and challenges. Statistical science. 2019; 34(2):301-316.
5. Janicki R. Properties of the beta regression model for small area estimation of proportions and application to estimation of poverty rates. Communications in Statistics-Theory and Methods. 2020; 49(9):2264-2284.
6. Rao JNK, Molina I. Small area estimation. John Wiley and Sons, 2015.
7. Morales D, Esteban MD, Pérez A, et al. A course on small area estimation and mixed models. Methods, theory and applications in R. 2021.
8. Tang X, Ghosh M, Ha N S, et al. Modeling random effects using global–local shrinkage priors in small area estimation. Journal of the American Statistical Association. 2018; 113(524): 1476-1489.
9. Pfeffermann D. New important developments in small area estimation. Statistical Science. 2013; 28(1):40-68.
10. Sugasawa S, Kubokawa T. Small area estimation with mixed models: a review. Japanese Journal of Statistics and Data Science. 2020; 3(2):693-720.
11. Datta GS, Ghosh M. Bayesian prediction in linear models: Applications to small area estimation. The Annals of Statistics. 1991; p.1748-1770.
12. Sinharay S, Stern HS. Posterior predictive model checking in hierarchical models. Journal of Statistical Planning and Inference. 2003; 111(1-2):209-221.
13. Chambers R, Chandra H, Salvati N, et al. Outlier robust small area estimation. Journal of the Royal Statistical Society: Series B (Statistical Methodology). 2014; 76(1):47-69.

14. Datta GS, Lahiri P. Robust hierarchical Bayes estimation of small area characteristics in the presence of covariates and outliers. Journal of Multivariate Analysis. 1995; 54(2):310-328.

15. Chambers RL. Outlier robust finite population estimation. Journal of the American Statistical Association. 1986; 81(396):1063-1069.

16. Chambers R, Tzavidis N. M-quantile models for small area estimation. Biometrika. 2006; 93(2):255-268.

17. Sinha SK, Rao JNK. Robust small area estimation. Canadian Journal of Statistics. 2009; 37(3):381-399.

18. Tang X , Ghosh M , Ha N S. Modeling Random Effects Using Global–Local Shrinkage Priors in Small Area Estimation. Journal of the American Statistical Association, 2018; 113.

19. Ghosh M, Lahiri P. Robust empirical Bayes estimation of means from stratified samples. Journal of the American Statistical Association. 1987; 82(400): 1153-1162.

20. Bell, William R, and Elizabeth T. Huang. "Using the t-distribution to deal with outliers in small area estimation." Proceedings of Statistics Canada Symposium. 2006.

21. Ghosh M, Maiti T, Roy A. Influence functions and robust Bayes and empirical Bayes small area estimation. Biometrika. 2008; 95(3):573-585.

22. Smith PA, Bocci C, Tzavidis N, et al. Robust estimation for small domains in business surveys. arXiv preprint arXiv:2006.01864, 2020.

23. Chakraborty A, Datta GS, Mandal A. Robust hierarchical Bayes small area estimation for the nested error linear regression model. International Statistical Review. 2019; 87:S158-S176.

24. Sinha SK. Robust small area estimation in generalized linear mixed models. Metron. 2019; 77(3):201-225.

25. Bertarelli G, Chambers R, Salvati N. Outlier robust small domain estimation via bias correction and robust bootstrapping. Statistical Methods and Applications. 2021; 30(1):331-357.

26. Jiang J, Rao JS. Robust small area estimation: An overview. Annual review of statistics and its application. 2020; 7:337-360.

27. Basu A, Harris IR, Hjort NL, Jones MC. Robust and efficient estimation by minimising a density power divergence. Biometrika. 1998; 85(3):549-559.

28. Ghosh A, Basu A. Robust estimation for independent non-homogeneous observations using density power divergence with applications to linear regression. Electronic Journal of statistics. 2013; 7:2420-2456.

29. Sugasawa S. Robust empirical Bayes small area estimation with density power divergence. Biometrika. 2020; 107(2):467-480.

30. Riani M, Atkinson A C, Corbellini A, Perrotta D. Robust regression with density power divergence: theory, comparisons, and data analysis. Entropy.2020; 22(4):399.

31. Kurisu D, Ishihara T,Sugasawa S. Adaptively robust small area estimation: Balancing robustness and efficiency of empirical Bayes confidence intervals. 2021. arXiv preprint arXiv:2108.11551.

32. Fujisawa H, Eguchi S. Robust parameter estimation with a small bias against heavy contamination. Journal of Multivariate Analysis. 2008; 99(9):2053–2081.

33. Warwick J, Jones MC. Choosing a robustness tuning parameter. Journal of Statistical Computation and Simulation. 2005; 75(7):581-588.

34. Basak S, Basu A, Jones MC. On the 'optimal'density power divergence tuning parameter. Journal of Applied Statistics. 2021; 48(3):536-556.

35. Sugasawa S, Yonekura S. On selection criteria for the tuning parameter in robust divergence. Entropy.2021; 23(9), 1147.

36. Hall P, Maiti T. On parametric bootstrap methods for small area prediction. Journal of the Royal Statistical Society: Series B (Statistical Methodology). 2006; 68(2):221-238.

37. Strawderman W E, Lehmann E L, Holmes S P. Elements of large-sample theory. New York, NY: Springer New York; 1998.
