# Supplementary material for: Robust small area estimation for unit level model with density power divergence
Source: PLoS One. 2023 Nov 16;18(11):e0288639. doi: 10.1371/journal.pone.0288639 (PMC10653428; doi:10.1371/journal.pone.0288639)
Supplement: Attachment — Submitted filename: Review Manuscript Number PONE-D-22-29623.pdf [file pone.0288639.s002.pdf]

REVIEW of the Manuscript Number PONE-D-22-29623

The authors discussed a new methodology for dealing with outliers in the Small Area Estimation framework. The aim of the paper is attractive; however, there are many sentences with grammatical and spelling mistakes - which can all be corrected with a careful editing review of the manuscript. Moreover, there are also several aspects throughout the manuscript that are not so clear. In addition to the poor attention to the editing style (font size reduction from page 6), these aspects make the manuscript a little bit hard to read. Moreover, I think that a more concrete and recent application is necessary. In my opinion, the authors should also revise the simulation section. I have the following comments:

- In my opinion, in the Introduction, the authors should better clarify the benefits of their method with respect to the alternatives. Indeed, on page 2, lines 83-91, the authors introduced the DPD method. However, they should provide a more precise explanation of the approach. Furthermore, they should better explain this approach's advantages concerning the approaches mentioned. In fact, for instance, the tuning constant in the Huber loss function allows controlling the trade-off between robustness and efficiency.
- In Section 5 the authors introduced the EB predictor. However, why do they not present the EBLUP, given the data used in the application and real data analysis?
- Simulation results are not so convincing on the superior performance of the proposed method. The differences in terms of performance among the proposed method and the alternatives are in several cases very small. I would suggest using relative measures. Moreover, the bias of σ_v^2 in scenarios $(0, v)$ and (v, e) using the SR approach are very different from those showed in the paper of Sinha and Rao, 2009. Could the authors check this and provide the code used for running simulations? Looking at the results of the RMD.opt I am not so convinced that the selection algorithm provides, in general, the best results (in some cases, i.e. in the last scenario, the results for variance components are not so good). I wonder if the selection algorithm is worth proposing. Indeed, the authors stated that "*The proposed RMD method is significantly better than the RML method, as we only need to select appropriate adjustment parameters*" but I am expecting that this should be automatic via the algorithm. The authors should clarify this point. Moreover, I would like to see a comparison with other methods, such as the MQ approach and the bias corrected version of the REBLUP (see Chambers R, Chandra H, Salvati N, et al. Outlier robust small area estimation. Journal of the Royal Statistical Society: Series B (Statistical Methodology. 2014; 76(1):47-69). Regarding the figures 1-12, firstly I do not fully understand why the authors use the *huberm* in the plots instead of the RML. Moreover, the authors contaminated the random terms up a proportion of 0.5. Is this share of contamination appropriate In this case, some other methods based on mixtures could be more appropriate. Could the authors better clarify this point?
- Regarding the real data analysis, I would see something more attractive than applying the method to such an old dataset. The authors should consider using their method to study a more recent and practical case. In this case the real data analysis seems like just an exercise.

Minor comments:

- Page 1, lines 2-3, "*In sampling estimation, due to the challenge of small sample or even no sample, small area estimation (SAE) method has received unanimous favor from statisticians.*", please add a reference. Also, for the examples below some other references should be added.
- "*[..] can solve the above difficulties*", it is not clear to which *difficulties* the authors refer.

- Page 1 line 9 *"[6] system describes the basic theory of small area;"* should be *"[6] describe the basic theory of small area estimation".* Many other typos are present throughout the manuscript. Please carefully revise the paper.
- Page 1 line 14, the authors stated that the model-based approach estimator has a smaller bias, but they do not specify with respect to which other approach. The direct estimates are usually unbiased. Please clarify.
- Page 2, from line 41 the authors stated some approaches to deal with the outliers, but no references of the various methods are given.
- *"In the basic unit level model, the errors of area random effect and model random effect are assumed to follow the normal distribution".* I think the authors could use individual and area-specific random effects to be clearer, instead of *area random effect and model random effect*
- The authors introduced the symbol v_i on page 2 line 58, but they do not define what this symbol refers to.
- Page 2, line 100, MDPD is not defined before.
- For equation 7,8,9 should be clear which components of θ refer
- From Section 4 the authors reduced the font size, and this does the manuscript very hard to read
- The section 5.3 should be moved after section 3. Indeed, the authors refer to "*above results*" that I suppose are those in section 3.
- In the simulation section formula of bias and MSE should be stated in the text. The symbol K in table 1 is not clear which indicates. The note at the end of the table is not specified elsewhere.
- The authors get editing help from someone with full professional proficiency in English. I noted a lot of multiple spelling and grammar errors.
