# Supplementary material for: Robust small area estimation for unit level model with density power divergence
Source: PLoS One. 2023 Nov 16;18(11):e0288639. doi: 10.1371/journal.pone.0288639 (PMC10653428; doi:10.1371/journal.pone.0288639)
Supplement: Attachment — Submitted filename: Response to Reviewers.docx [file pone.0288639.s003.docx]

**Response to Reviewers**

I am very glad to receive comments from reviewers, who have provided many meaningful suggestions for further improvement of this paper. In view of the amendments of the articles, this article made the following changes:

In view of the reviewer's main comments, this paper has been modified as follows:

1. For the first opinion, in the introduction part, the application background of DPD method is introduced in detail, and the advantages of this method compared with other methods are described, and some references are added. The changes in this section are between lines 90 and 108.
2. In section 5, the EBLUP can be obtained by substituting the estimated parameter values into the EBP, which is explained in section 5.
3. As for the third comments, the simulation part of the paper has been modified in detail. First, the Bias and MSE in the table become relative Bias and relative MSE. The parameter selection program is optimized and the values in the table are modified. This part is mainly in the numerical simulation part of the paper. It can be seen from the simulation results that the optimal adjustment parameters selected after modification can achieve the optimal estimation effect.
4. In the application of actual data, although the data used in this paper is relatively old, it can also illustrate the effect of the method proposed in this paper.

For minor modification suggestions, the main contents are as follows:

- In line 14, the difference between direct and model-based estimates is explained.
- Modify "area random effect and model random effect" to" individual and area-specific random effect".
- In line 156, subheading 3.1 has been added
- In the definition of DPD, the meaning of parameter θ is given.
- Adjust the content of Section 5.3 to Section 3.2.
- Formula 16 and 17 in line 280 are numbered
- The section 5 explains the relationship between EBP and EBLUP.
- In the simulation part, the expression formulas of relative bias deviation and relative MSE are given, and the performance results of relative MSE are shown in the simulation table.
- Huberm methods have been changed to RML methods in the simulation part of the images and tables.
- In the discussion section, the applicability of other parameter selection algorithms and other robust estimation methods is pointed out.
- Some references have been added.
- Some inaccuracies in grammar and expression have been corrected.
